# Isoflurane and Sevoflurane Induce Severe Hepatic Insulin Resistance in a Canine Model

**DOI:** 10.1371/journal.pone.0163275

**Published:** 2016-11-01

**Authors:** Stella P. Kim, Josiane L. Broussard, Cathryn M. Kolka

**Affiliations:** Diabetes and Obesity Research Institute, Cedars-Sinai Medical Center, Los Angeles, CA, 90048, United States of America; University of Melbourne, AUSTRALIA

## Abstract

**Introduction:**

Anesthesia induces insulin resistance, which may contribute to elevated blood glucose and adverse post-operative outcomes in critically ill patients, and impair glycemic control in surgical patients with diabetes. However, little is known about the mechanisms by which anesthesia impairs insulin sensitivity. Here we investigate the effects of anesthesia on insulin sensitivity in metabolic tissues.

**Methods:**

Hyperinsulinemic-euglycemic clamps were performed in 32 lean (control diet; n = 16 conscious versus n = 16 anesthetized) and 24 fat-fed (6 weeks fat-feeding; n = 16 conscious versus n = 8 anesthetized) adult male mongrel dogs in conjunction with tracer methodology to differentiate hepatic versus peripheral insulin sensitivity. Propofol was administered as an intravenous bolus (3mg/kg) to initiate anesthesia, which was then maintained with inhaled sevoflurane or isoflurane (2–3%) for the duration of the procedure.

**Results:**

Anesthesia reduced peripheral insulin sensitivity by approximately 50% in both lean and fat-fed animals as compared to conscious animals, and insulin action at the liver was almost completely suppressed during anesthesia such that hepatic insulin sensitivity was decreased by 75.5% and; 116.2% in lean and fat-fed groups, respectively.

**Conclusion:**

Inhaled anesthesia induces severe hepatic insulin resistance in a canine model. Countermeasures that preserve hepatic insulin sensitivity may represent a therapeutic target that could improve surgical outcomes in both diabetic and healthy patients.

## Introduction

Anesthesia induces insulin resistance [1], though little is known about the causal mechanism or specific tissues involved. Both isoflurane and sevoflurane impair glucose tolerance, which worsen post-operative outcomes, not only by promoting hyperglycemia, but also by enhancing catabolism and muscle wasting [1]. A previous study demonstrated that sevoflurane impairs glucose tolerance and insulin secretion [2]. Also, isoflurane decreases lipolysis, insulin sensitivity, and increases glycemia both through increased glucose production by the liver and decreased peripheral glucose utilization [3]. Glycemic control, mediated by both production and uptake of glucose, is particularly important in surgery, where poor control is associated with adverse outcomes post-surgery [4]. Understanding the effects of anesthesia on various tissues is therefore important in promoting better surgical outcomes.

In healthy, non-diabetic animals and humans, insulin suppresses hepatic glucose production and stimulates peripheral glucose uptake [5–7]. Our previous findings in the canine model have shown that the liver is particularly susceptible to impairments in insulin sensitivity (S_I_) induced by a high fat diet, as 12 weeks of fat feeding in this study did not cause a significant reduction in peripheral insulin sensitivity, whereas hepatic insulin sensitivity was completely impaired [8]. Here we extend these findings by demonstrating that the liver may be especially vulnerable to anesthesia-induced insulin resistance.

## Methods

### Animals

Male mongrel hounds (25-30kg) were purchased from Antech Inc (Barnhart, MO) and were housed in the University of Southern California Medical School Vivarium or Cedars-Sinai Comparative Medicine facility under controlled kennel conditions (12h light:12h dark). Animals were fasted overnight before the morning of the experiment. Dogs were used for experiments only if judged to be in good health as determined by visual observation, body weight, hematocrit and body temperature, and all efforts were made to minimize suffering. Protocols were conducted in conformity with the Public Health Service (PHS) Policy on Humane Care and Use of Laboratory Animals, and approved by the University of Southern California (USC) Institutional Animal Care and Use Committee, or the Cedars-Sinai Medical Center Institutional Animal Care and Use Committee, as appropriate. There was no significant effect of location on results (data not shown). Hyperinsulinemic-euglycemic clamps were performed in 32 lean (control diet; n = 16 conscious, 28.4±1.1kg, versus n = 16 anesthetized, 28.5±0.7kg) and 24 fat-fed adult male mongrel dogs (6 weeks fat-feeding; n = 16 conscious 30.9±1.4kg versus n = 8 anesthetized, 28.2±1.0kg), using tracer methodology to differentiate hepatic and peripheral insulin sensitivity, as previously described [9]. Some data from these experiments has been previously published [8, 10, 11], here we compare the effects of anesthesia on insulin sensitivity for the first time.

### Anesthesia

Dogs were sedated with acepromazine maleate (Prom-Ace, Aueco, Fort Dodge, IA; 0.22mg/kg) and atropine sulfate (Western Medical, Arcadia, CA; 0.11 mL/kg). Anesthesia was induced with intravenous administration of propofol (Western Medical, Arcadia, CA; 6mg/kg) and maintained with inhaled isofluorane or sevoflurane (Western Medical, Arcadia, CA). Dogs were placed on heating pads to maintain body temperature. Saline was infused into the cephalic vein and maintained at a slow drip throughout the experiment. Indwelling catheters were placed into both the left femoral artery and vein for sampling. Blood pressure, heart rate, O_2_ saturation and CO_2_ were monitored continuously. At the conclusion of these experiments, animals were euthanized with an overdose of sodium pentobarbital (Eutha-6, Western Medical; 65mg/kg).

### Hyperinsulinemic euglycemic clamp (EGC)

Conscious animals were mildly restrained in a Pavlov harness. Infusions were made into cephalic or saphenous veins, and blood samples were taken from femoral vessels (in anesthetized animals) or cephalic veins (in conscious animals) every 10–15 minutes throughout the study. A primed tracer infusion of [3-^3^H]glucose (priming dose, 25μCi; infusion, 0.25μCi/min; DuPont-NEN, Boston, MA) was administered, and exogenous glucose labeled with [3-^3^H]glucose (2.7μCi/g glucose) was infused at variable rates to clamp plasma glucose to the basal level throughout the entire experimental period. Somatostatin was infused to inhibit endogenous insulin secretion (1ug/min/kg; Bachem, Torrance CA). After 120–180 minutes of tracer infusion, insulin was infused at 0.75–1.0mU/min/kg (Novo Nordisk, Bagsvaerd, Denmark) for the remainder of the study.

### Assays

Blood samples were collected in microtubes pre-coated with lithium-heparin (Becton Dickinson, Franklin Lakes, NJ) containing 50μL EDTA (Sigma Chemicals, St Louis, MO). Blood samples were centrifuged immediately, and the supernatant was assayed for glucose with a YSI 2700 autoanalyzer (Yellow Springs Instrument Co., Yellow Springs, OH), then frozen at -20°C. Plasma insulin was measured with an ELISA for dog plasma (Alpco, Salem, NH). Samples for measuring [3-^3^H]glucose were deproteinized with barium hydroxide and zinc sulfate, evaporated to remove radiolabeled water, and counted in Ready Safe scintillation fluid (Beckman Instruments, Fullerton, CA). Tracer-determined whole body glucose disposal (Rd) and endogenous glucose production (EGP) were calculated using Steele’s equation modified for use with labeled glucose infusion [9] after smoothing plasma glucose and tracer data by optimal segments. Steady state was defined as the final thirty minutes of the hyperinsulinemic euglycemic clamp. Peripheral insulin sensitivity is calculated from the peripheral insulin action (ΔRd), the change in plasma insulin (ΔIns), and the glucose concentration at steady state (Gluc_SS_) by the equation ΔRd/(ΔInsxGluc_SS_). Similarly, hepatic insulin sensitivity is calculated using the hepatic insulin effect (ΔEGP), with the equation ΔEGP/(ΔInsxGluc_SS_) (9).

### Statistical analyses

Experimental data are shown as mean ± standard error of the mean (SEM). Statistical analyses were performed with paired or unpaired Student’s *t* tests or two-way ANOVAs with Tukey’s pairwise comparisons, as appropriate (GraphPad Prism version 5.04 for Windows, GraphPad Software, San Diego, CA). Differences were considered statistically significant when *p*<0.05.

## Results

**Fig 1:**
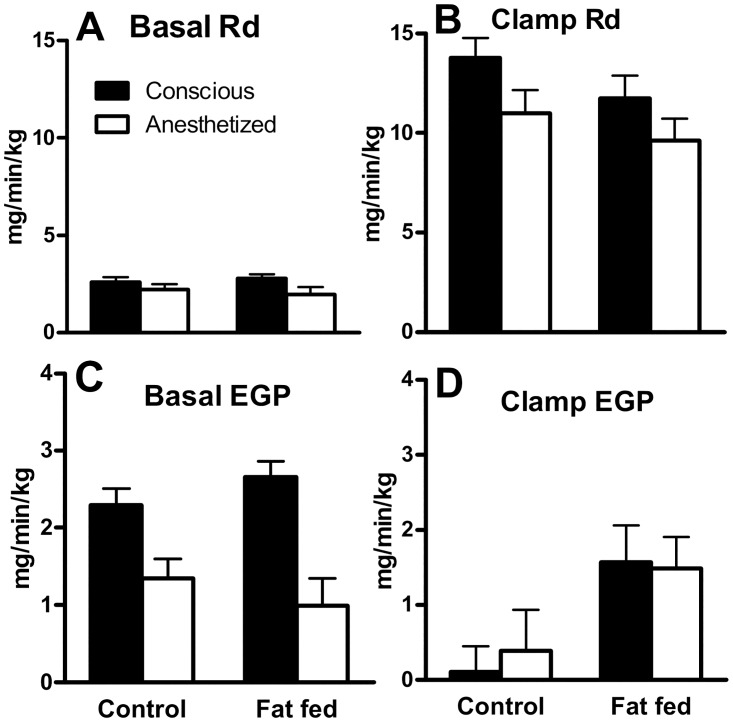
Effects of anesthesia and diet on measures of glucose metabolism. Rd (A and B) and EGP (C and D) were assessed under conscious (black bars) and anesthetized (white bars) conditions in lean and fat fed animals (mean±SEM). Assessments were taken under low insulin (BASAL) conditions (A and C), and under the hyperinsulinemia induced by the CLAMP (B and D).

Under basal conditions, Rd was low and similar in all groups (Fig 1A). Insulin stimulated glucose disposal, as evidenced by increased Rd under clamp conditions. However, anesthesia significantly decreased Rd (Fig 1B) resulting in a 20% suppression of insulin-mediated glucose disposal compared to conscious animals. Although there was no significant effect of diet on basal glucose production (Fig 1C), glucose production was significantly reduced by anesthesia. Insulin normally suppressed glucose production, as observed in Fig 1D, however this effect was lost with fat feeding. Interestingly, this effect was completely lost under anesthesia, such that even in lean animals there was no insulin-mediated suppression of endogenous glucose production. There was no interaction of diet and anesthesia on any of the measured variables. We also assessed the metabolic clearance rate of insulin, which is calculated by dividing the insulin infusion rate by the insulin levels at steady state during the clamp, and there was no effect of either diet or anesthesia (LEAN: Conscious 2.8±0.3, Anesthetized 2.6±0.2. FAT FED: Conscious 2.3±0.3 Anesthetized 2.2±0.1).

**Fig 2:**
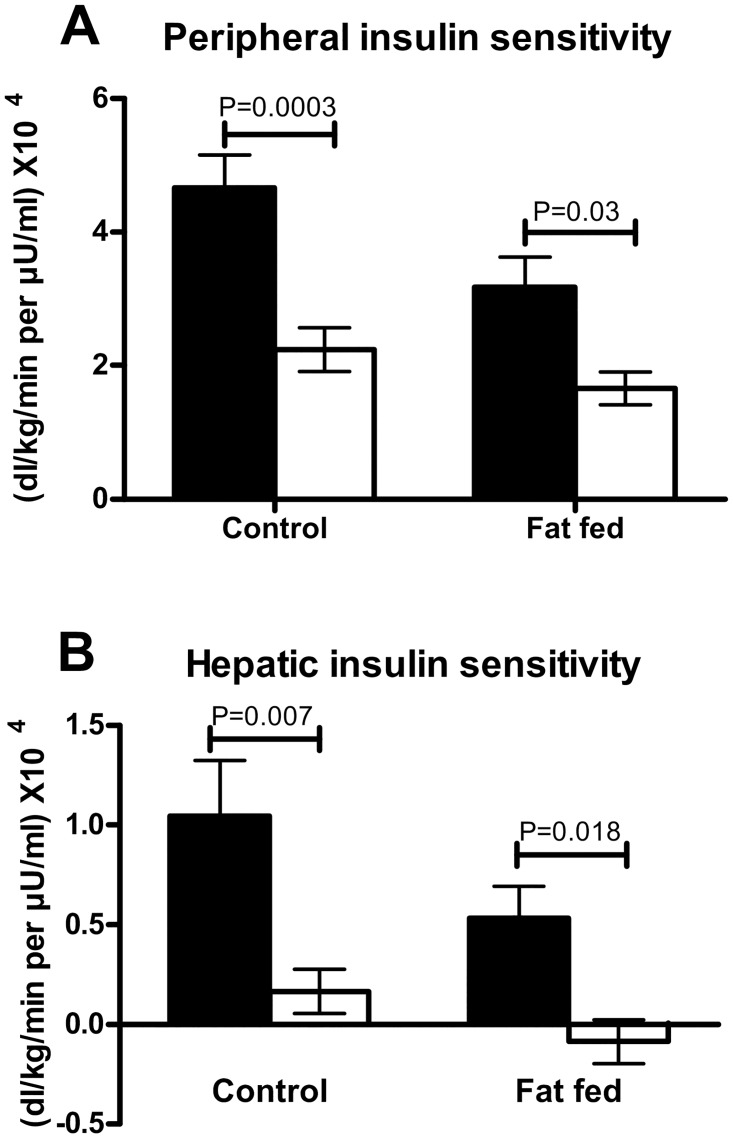
Insulin sensitivity with and without anesthesia. (A) Peripheral and (B) hepatic insulin sensitivity, as calculated from Rd and EGP, respectively was assessed under conscious (black bars) and anesthetized (white bars) conditions in lean and fat-fed animals (mean ± SEM).

Peripheral insulin sensitivity during anesthesia was 50% of that in conscious animals, indicating peripheral insulin resistance (52.0% and 47.9% reduction in lean and fat-fed, respectively). Anesthesia was also associated with a reduction in insulin sensitivity in the liver as compared to conscious animals (75.5% and 116.2% reduction in lean and fat-fed, respectively; Fig 2), indicating severe hepatic insulin resistance (raw data in S1 Table).

In a subset of lean animals, we detected no difference between the two anesthetic agents on peripheral or hepatic insulin sensitivity (peripheral S_I_: isoflurane: 2.3±0.7, n = 10 versus sevoflurane: 2.2±0.4, n = 6; dl/kg/min per μU/ml x10^4^; p = ns; hepatic S_I_: isoflurane: 0.3±0.2 versus sevoflurane: 0.4±0.2 dl/kg/min per μU/ml x10^4^, p = ns).

## Discussion

The effects of a high fat diet on body weight and insulin sensitivity have been reported previously [8], and it is generally known that anesthesia can induce insulin resistance [1]. Anesthesia has been shown to impair pancreatic release of insulin [1, 12], and peripheral insulin resistance is also noted. Here we show that the S_I_ in the periphery is reduced by approximately 50% with anesthesia, whereas liver S_I_ is almost completely impaired, resulting in a higher level of glucose production by the liver, which may contribute to hyperglycemia under anesthesia.

The hyperinsulinemic clamp allows the separate analysis of glucose removal by the periphery (Rd) and endogenous production (EGP) by the liver. Here we demonstrate that glucose utilization under basal conditions is not significantly altered by either diet or anesthesia, however the normal effects of insulin to increase Rd are impaired by anesthesia. In this study, we observed no effect of diet to induce peripheral insulin resistance, as previously reported [8]. EGP, driven primarily by the liver, was suppressed under anesthesia. It is possible that reduced glucose requirements under anesthesia due to reduced motor activity requirements and tonic muscle tone may contribute to a reduction in glucose production, however we did not observe a commensurate drop in Rd that would support this hypothesis. Further, the ability of insulin to suppress EGP under both fat feeding and anesthesia conditions was reduced. Thus, anesthesia itself impairs hepatic glucose metabolism.

In the present study, assessments of S_I_ occurred after animals were maintained on inhalant anesthesia for more than 5 hours. While propofol can cause acute insulin resistance [13], its effects are short-lived, lasting approximately 3–5 minutes, therefore the effects of anesthesia on S_I_ are likely driven by inhaled anesthesia. Propofol itself can increase hepatic blood flow and liver oxygen consumption [14], and while some inhaled anesthetics impair liver blood flow, isoflurane seems to preserve volumetric blood flow in the liver microcirculation [15]. Thus, it is unlikely that the observed changes in liver metabolism are due to changes in liver blood flow, which is a major consideration in assessing perioperative risk [16]. Studies investigating the effects of inhaled anesthesia on liver enzymes found that although isoflurane and sevoflurane did not have an acute effect, [17] liver enzymes were elevated 2–7 days after recovery, which may indicate that liver function was affected in a subclinical manner by anesthetic exposure [17], and may be the cause of the metabolic changes observed.

Impaired metabolism has previously been detected with anesthesia, including decreased protein synthesis, reduced plasma lipid levels, and reduced peripheral glucose uptake with increased glucose production [3], which would contribute to hyperglycemia. Hyperglycemia and poor glycemic control during surgery is associated with negative clinical outcomes in diabetic patients [4]. While the effect of anesthesia in diabetic and pre-diabetic patients is a major concern, our results indicate that anesthesia may also impair glucose homeostasis in otherwise healthy, lean individuals, and suggests the liver may be a primary site of impaired metabolism. A recent review stated that while aggressive insulin therapy post-operatively can improve morbidity and mortality rates, similar results can be attained by using pre-operative nutrition strategies to minimize insulin resistance [18]. Our studies were performed in fasted animals, and it is therefore possible that a pre-operative carbohydrate intervention may alter systemic or hepatic insulin sensitivity, and thus improve metabolism during the surgery.

We have used the canine as a model of human obesity and insulin resistance to show that even modest increases in body weight induced by a high fat diet are associated with development of insulin resistance [8, 19]. A review concluded that the metabolic syndrome does not occur dogs as it does in humans: although the development of visceral adiposity and obesity-induced insulin resistance does occur, it is not associated with atherosclerosis, and only mild changes in plasma lipid profile are detected [20]. Thus, it seems unlikely that a prolonged exposure to a high fat diet alone will eventually result in diabetes in the canine, particularly in the absence of any pancreatic defect[21]. However, recent GWAS studies of human populations have demonstrated that a healthy pancreas can adequately compensate for insulin resistance, but any beta cell defect predisposes an individual towards development of diabetes [22]. Therefore, our obese canine model represents an insulin resistant phenotype, but not frank diabetes. We show that anesthesia itself can alter glucose metabolism in liver and peripheral tissues in both lean and insulin resistant states. Supporting this finding, there have been documented instances of hyperglycemia under anesthesia in diabetic [23] and lean patients [24]; this latter study also demonstrated a reduction in the rate of glucose appearance and clearance under anesthesia in humans. Thus, our results are highly relevant to humans, such that anesthesia alters both glucose metabolism and insulin sensitivity even in healthy lean individuals.

In conclusion, we have demonstrated that inhaled anesthesia used in routine surgeries causes severe hepatic insulin resistance and altered hepatic glucose metabolism in canines. Insulin resistance may contribute to elevated glucose levels under anesthesia, which is associated with increased mortality in critically ill patients and may complicate glycemic control in diabetic surgical patients. Further studies are needed to assess the full clinical implications of these findings as well as determine strategies to mitigate anesthesia-induced insulin resistance during surgery in both diabetic and healthy patients.

## Supporting Information

S1 Table Measures of insulin, glucose and glucose metabolism. Supplemental data showing values of insulin, glucose metabolism and glucose levels at basal (0min, before insulin infusion) and at steady state (180min) under hyperinsulinemic conditions.
(PDF)
