# Supplementary material for: Isoflurane and Sevoflurane Induce Severe Hepatic Insulin Resistance in a Canine Model
Source: PLoS One. 2016 Nov 1;11(11):e0163275. doi: 10.1371/journal.pone.0163275 (PMC5089720; doi:10.1371/journal.pone.0163275)
Supplement: S1 Table — Supplemental data showing values of insulin, glucose metabolism and glucose levels at basal (0min, before insulin infusion) and at steady state (180min) under hyperinsulinemic conditions. (PDF) [file pone.0163275.s001.pdf]

ANESTHETIZED

LEAN	DOG1	DOG2	DOG3	DOG4	DOG5	DOG6	DOG7	DOG8	DOG9	DOG10	DOG11	DOG12	DOG13	DOG14	DOG15	DOG16
**WEIGHT**	27.1	27.3	23.5	29	26	29	25.9	28	26.1	31.9	31.2	27.4	30.6	34.8	28.2	29.95
Ins Basal (µU/ml)	8.20	8.71	2.86	13.60	8.01	4.84	7.48	11.00	6.12	4.80	4.10	2.78	2.78	6.59	5.11	3.45
Ins 0 min(µU/ml)	14.07	14.44	13.11	11.62	11.78	15.43	17.07	16.47	18.36	14.22	15.00	9.71	5.73	13.69	11.23	15.21
ins 180 min (µU/ml)	79.68	101.18	76.37	75.55	74.49	73.23	92.54	123.00	162.07	99.94	88.39	64.73	48.91	92.71	55.63	77.70
insulin 0min (pM)	84.39	86.67	78.64	69.70	70.66	92.55	102.41	98.80	110.14	85.31	89.99	58.26	34.36	82.12	67.40	91.24
insulin 180 min (pM)	478.07	607.08	458.20	453.28	446.92	439.37	555.26	737.97	972.45	599.66	530.34	388.40	293.46	556.24	333.76	466.18
Rd 0 min (mg/min/kg)	2.44	2.09	4.80	3.12	5.63	2.65	2.49	2.32	1.82	2.03	1.53	1.55	2.75	0.93	2.02	2.65
Rd 180 min	5.15	4.23	21.36	16.05	17.12	11.91	9.38	10.29	8.69	13.50	8.39	9.51	12.18	6.00	14.21	7.86
EGP 0 min (mg/min/kg)	1.69	0.78	1.81	1.19	4.60	1.66	1.43	1.25	0.73	1.12	0.55	0.18	1.83	0.21	1.43	1.05
EGP 180 min	0.04	-0.07	5.59	1.35	2.68	1.19	0.16	1.88	-0.63	0.75	-0.53	0.00	1.04	-0.62	-1.72	-4.83
GINF 0 min (mg/min/kg)	0.67	1.23	2.86	1.53	0.77	0.97	0.98	0.91	1.11	0.78	0.69	1.33	0.89	0.78	0.24	1.36
GINF 180min	5.16	4.39	15.46	14.78	14.32	10.90	9.15	8.63	8.63	12.54	9.11	9.63	11.15	6.72	16.29	12.73
Glucose 0min (mg/dl)	95.46	99.54	94.71	92.99	97.40	99.14	102.43	91.10	96.13	95.73	78.86	95.46	93.85	99.80	110.30	93.11
Glucose 180min	104.46	102.10	96.88	98.26	102.40	96.61	97.39	93.25	85.61	96.40	94.13	97.13	88.49	94.10	112.36	96.28

FAT FED	DOG1	DOG2	DOG3	DOG4	DOG5	DOG6	DOG7	DOG8
**WEIGHT**	31.1	30.5	31.5	28.5	27.2	27	24.1	25.5
Ins -180min (µU/ml)	11.72	7.98	7.39	7.86	7.56	8.45	0.00	2.14
Ins 0 min (µU/ml)	16.28	19.92	12.18	14.85	11.48	18.49	9.82	16.10
ins 180 min (µU/ml)	84.57	111.30	80.83	108.85	95.27	97.77	72.85	114.46
insulin 0 min (pM)	97.71	119.55	73.06	89.10	68.91	110.95	58.92	96.61
insulin 180 min (pM)	507.44	667.80	485.00	653.08	571.61	586.62	437.12	686.79
Rd 0 min (mg/min/kg)	0.02	3.18	1.68	2.00	2.83	2.90	2.18	0.87
Rd 180 min	8.45	14.07	10.29	10.88	4.47	6.70	9.01	12.93
EGP 0 min (mg/min/kg)	-0.66	1.16	1.63	1.08	2.23	1.75	1.04	-0.30
EGP 180 min	0.21	3.57	1.65	2.66	0.14	1.28	0.65	1.73
GINF 0 min (mg/min/kg)	2.04	1.85	0.32	0.70	0.67	0.94	1.07	1.14
GINF 180 min	9.34	8.10	8.65	8.41	4.67	5.38	8.44	11.04
Glucose 0 min (mg/dl)	96.44	97.44	98.61	96.86	94.40	100.13	99.76	95.91
Glucose 180 min	94.63	99.83	102.13	94.04	90.06	94.95	98.98	87.35

CONSCIOUS

CONTROL	Dog1	Dog2	Dog3	Dog4	Dog5	Dog6	Dog7	Dog8	Dog9	Dog10	Dog11	Dog12	Dog13	Dog14	Dog15	Dog16
**WEIGHT**	29.7	33.2	31.7	26.5	20.9	26.2	23.8	28.7	26.1	29.7	33.4	26.6	22.3	27.0	33.0	35.5
Ins basal (µU/ml)	2.38	3.07	3.90	10.88	5.48	3.08	2.69	5.80	12.36	18.10	4.39	5.80	18.88	13.80	10.03	27.21
ins 180 (µU/ml)	30.63	30.70	30.61	38.33	29.40	23.71	33.08	37.19	94.91	138.38	73.54	70.12	76.70	86.46	58.83	98.68
Ins basal (pM)	14.31	18.39	23.43	65.31	32.87	18.46	16.14	34.80	74.14	108.63	26.32	34.81	113.26	82.80	60.18	163.25
insulin 180 (pM)	183.78	184.20	183.68	229.98	176.40	142.25	198.50	223.13	569.45	830.25	441.23	420.73	460.20	518.78	352.97	592.09
Rd basal (mg/min/kg)	1.00	2.15	4.01	3.48	3.26	3.30	2.51	1.61	3.82	2.71	2.68	2.16	0.64	3.58	3.15	1.62
Rd 180	15.64	9.76	13.59	11.23	11.98	12.18	11.57	11.06	17.57	13.68	23.79	16.48	9.37	19.26	12.68	10.63
EGP basal (mg/min/kg)	0.74	2.13	3.84	3.00	2.63	3.08	2.62	1.50	2.82	2.51	2.34	2.09	0.68	2.09	3.05	1.59
EGP 180	0.56	0.81	1.82	0.83	0.06	-2.34	1.02	-0.90	0.39	0.99	1.30	-2.74	-1.68	-0.35	1.32	0.75
																0.00
GINF basal (mg/min/kg)	0.00	0.00	0.00	0.00	0.00	0.00	0.00	0.00	0.00	0.00	0.00	0.00	0.00	0.00	0.00	0.00
GINF 180	14.78	9.09	11.98	10.53	11.96	14.03	10.36	11.48	16.44	11.24	22.85	19.11	11.14	18.97	10.98	9.58
Glucose basal (mg/dl)	96.61	98.53	97.04	102.22	101.99	96.34	100.17	94.62	94.20	99.33	89.10	95.13	102.88	97.86	99.83	100.38
Glucose 180	98.21	98.79	93.75	100.64	97.85	91.96	99.76	94.83	89.71	88.88	93.89	103.84	106.13	95.88	89.23	95.43

FAT-FED	Dog1	Dog2	Dog3	Dog4	Dog5	Dog6	Dog7	Dog8	Dog9	Dog10	Dog11	Dog12	Dog13	Dog14	Dog15	Dog16
**WEIGHT**	37.2	37.2	35.5	27.7	21.7	27.9	25.5	31.2	28.4	33.2	33.3	25.9	22.7	30.6	37.5	38.3
Ins basal (µU/ml)	10.20	14.04	8.67	11.10	4.25	4.23	4.66	5.39	14.86	21.14	24.42	7.43	29.43	18.47	18.47	32.41
ins 180 (µU/ml)	33.63	47.70	48.06	34.36	35.49	34.58	32.64	34.41	136.22	225.42	123.45	73.17	105.34	98.73	73.02	96.90
Ins basal (pM)	61.19	84.21	52.04	66.59	25.52	25.38	27.96	32.33	89.14	126.86	146.50	44.59	176.58	110.79	110.83	194.43
insulin 180 (pM)	201.80	286.18	288.38	206.15	212.95	207.47	195.86	206.48	817.30	1352.50	740.72	439.01	632.05	592.37	438.13	581.40
Rd basal (mg/min/kg)	2.60	2.30	2.88	2.58	1.74	2.74	2.54	2.77	1.96	2.58	3.39	5.31	3.18	3.33	2.15	2.80
Rd 180	12.12	7.49	12.27	8.31	9.16	13.26	5.61	11.64	16.43	17.81	16.91	17.72	6.87	18.36	7.58	6.17
EGP basal (mg/min/kg)	2.41	2.13	2.81	2.51	1.82	2.37	2.31	2.60	1.75	2.75	3.03	5.37	3.04	2.83	2.23	2.57
EGP 180	3.58	-0.48	0.51	1.13	0.09	0.88	-0.21	1.21	0.55	6.84	3.86	3.93	0.04	0.98	1.36	0.84
GINF basal (mg/min/kg)	0.00	0.00	0.00	0.00	0.00	0.00	0.00	0.00	0.00	0.00	0.00	0.12	0.00	0.37	0.00	0.00
GINF 180	8.55	8.41	11.72	7.84	9.12	12.30	6.01	10.35	15.42	10.76	13.71	13.73	9.18	17.21	6.35	5.83
Glucose basal (mg/dl)	101.65	99.02	95.64	95.23	96.20	95.15	98.94	96.60	95.41	94.58	96.05	92.89	98.04	86.86	95.23	94.60
Glucose 180	95.46	95.48	92.41	99.08	94.70	102.64	96.04	96.54	93.00	88.33	97.90	96.81	92.79	97.51	101.15	110.50

Table: Supplemental data showing values of insulin, glucose metabolism and glucose levels at basal (0min, before insulin infusion) and at steady state (180min) under hyperinsulinemic conditions.
